# Primary Burkitt Lymphoma of the Thyroid Gland With Underlying Hashimoto’s Thyroiditis: A Case Report

**DOI:** 10.7759/cureus.95155

**Published:** 2025-10-22

**Authors:** Dhruvin Shah, Narendra Hirani, Ajeet K Khilnani, Rashmi Sorathiya, Nikita Modh

**Affiliations:** 1 Otolaryngology - Head and Neck Surgery, Gujarat Adani Institute of Medical Sciences, Bhuj, IND; 2 Pathology, Gujarat Adani Institute of Medical Sciences, Bhuj, IND

**Keywords:** b-cell neoplasm, extra-nodal lymphoma, non-hodgkin’s lymphomas, primary burkitt lymphoma, hashimoto’s thyroiditis

## Abstract

Primary Burkitt lymphoma of the thyroid is an exceptionally rare, highly aggressive B-cell malignancy. We present the case of a 30-year-old male with a rapidly progressive anterior neck swelling. Imaging revealed a large left thyroid mass with cervical lymphadenopathy, and fine needle aspiration cytology was suspicious for malignancy. Total thyroidectomy was performed, and histopathology and immunohistochemistry confirmed Burkitt lymphoma. The patient received three cycles of R-CHOEP (rituximab, cyclophosphamide, vincristine, adriamycin, etoposide, and prednisolone) chemotherapy, and follow-up positron emission tomography-computed tomography after three months demonstrated complete remission. This case highlights the importance of early diagnosis and prompt chemotherapy in achieving favorable outcomes for this rare entity.

## Introduction

Burkitt lymphoma (BL) is an extra-nodal lymphoma characterized by a rapid growth rate, aggressive clinical course, and characteristic morphologic, immunophenotypic, and molecular genetic findings [1]. The head and neck region is the second most common site of BL, with most cases being found in the nasopharynx, Waldeyer’s ring, and sinuses [2,3]. BL is a highly aggressive B-cell neoplasm, typically associated with a myelocytomatosis (MYC) oncogene translocation, and is characterized by its rapid growth and high mitotic index [4]. Although the thyroid gland normally lacks lymphoid tissue, chronic inflammatory conditions such as Hashimoto's thyroiditis (HT) can promote lymphoid infiltration, creating an environment conducive to lymphoma development [5]. HT is most frequently linked to mucosa-associated lymphoid tissue lymphoma and diffuse large B-cell lymphoma, while its association with BL remains exceptionally rare [6]. BL in the thyroid gland is extremely rare, and only a limited number of cases are reported in the literature. So here we report a case of primary BL of the thyroid gland with underlying changes of HT.

## Case presentation

A 30-year-old male from a lower socioeconomic background presented to the otorhinolaryngology outpatient department of a tertiary care hospital in western Gujarat. He complained of anterior neck swelling for one month. The swelling was insidious in onset and rapidly progressive. It moved with deglutition but did not move with protrusion of the tongue. It was associated with occasional pain and generalized weakness. There were no symptoms of hypo or hyperthyroidism. He had no respiratory or swallowing issues. There was no history of fever, weight loss, or any history of thyroid medication.

**Figure 1:**
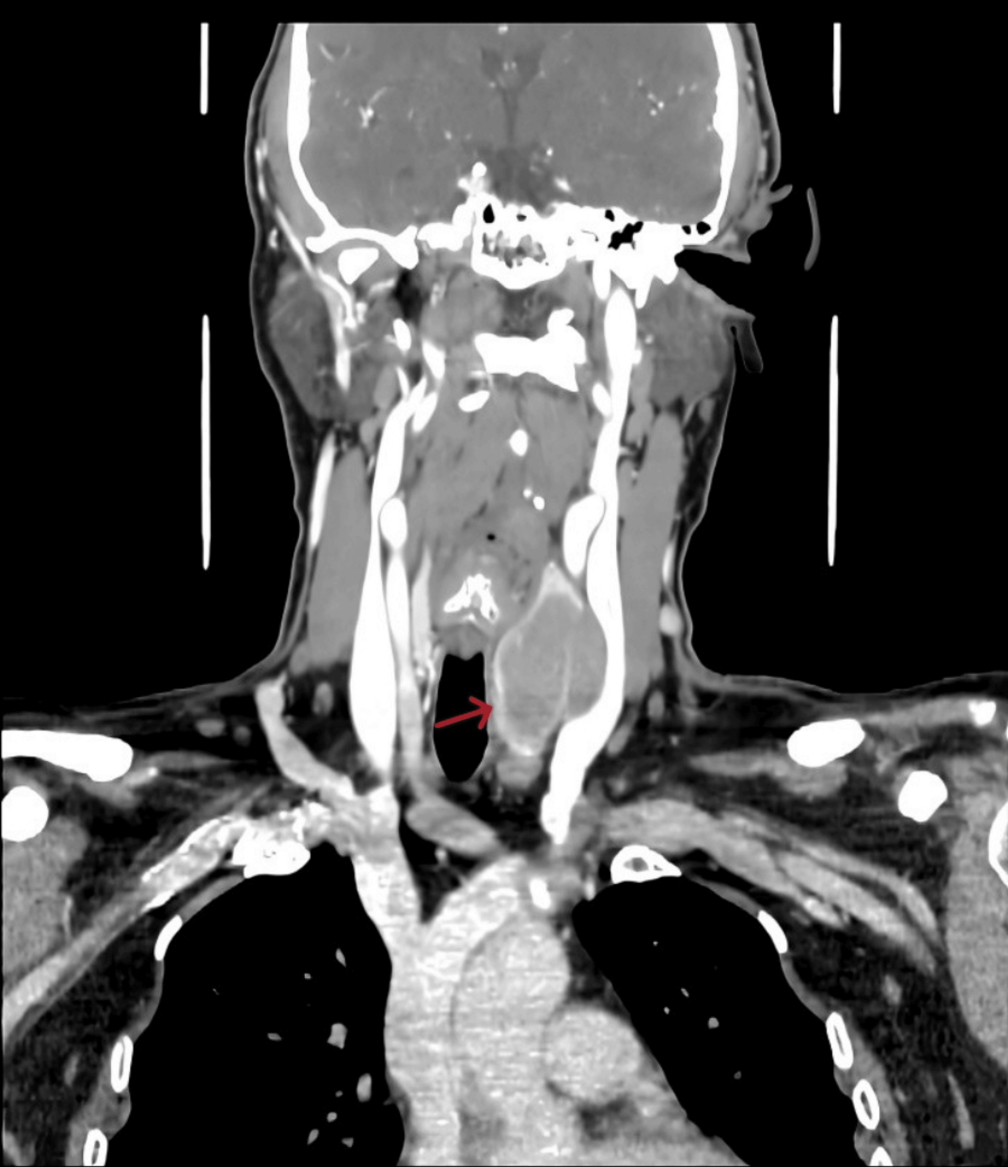
CECT neck coronal section showing a well-defined lobulated exophytic soft tissue lesion involving the left lobe and isthmus of the thyroid (red arrow) CECT: Contrast-enhanced computed tomography.

Ultrasonography showed a left-sided thyroisthmal cyst and a large left thyroid nodule with left cervical lymphadenopathy. Thyroid function tests were within normal limits. Fine needle aspiration cytology was suggestive of atypical cells, highly suspicious of malignancy. The two possibilities considered were undifferentiated carcinoma of the thyroid and a lymphoproliferative disorder. Based on these investigations and in view of suspicion of malignancy, contrast-enhanced computed tomography of the neck was performed, which revealed a well-defined lobulated exophytic soft tissue lesion involving the left lobe and isthmus of the thyroid (Figure 1). It measured 32 mm × 45 mm in the axial plane with a cranio-caudal extension of 46 mm. The lesion showed minimal enhancement on the post-contrast study. Malignancy could not be ruled out. Multiple enlarged lymph nodes were observed at levels Ia, Ib, II, III, and IV on both sides and level VI on the left side.

**Figure 2:**
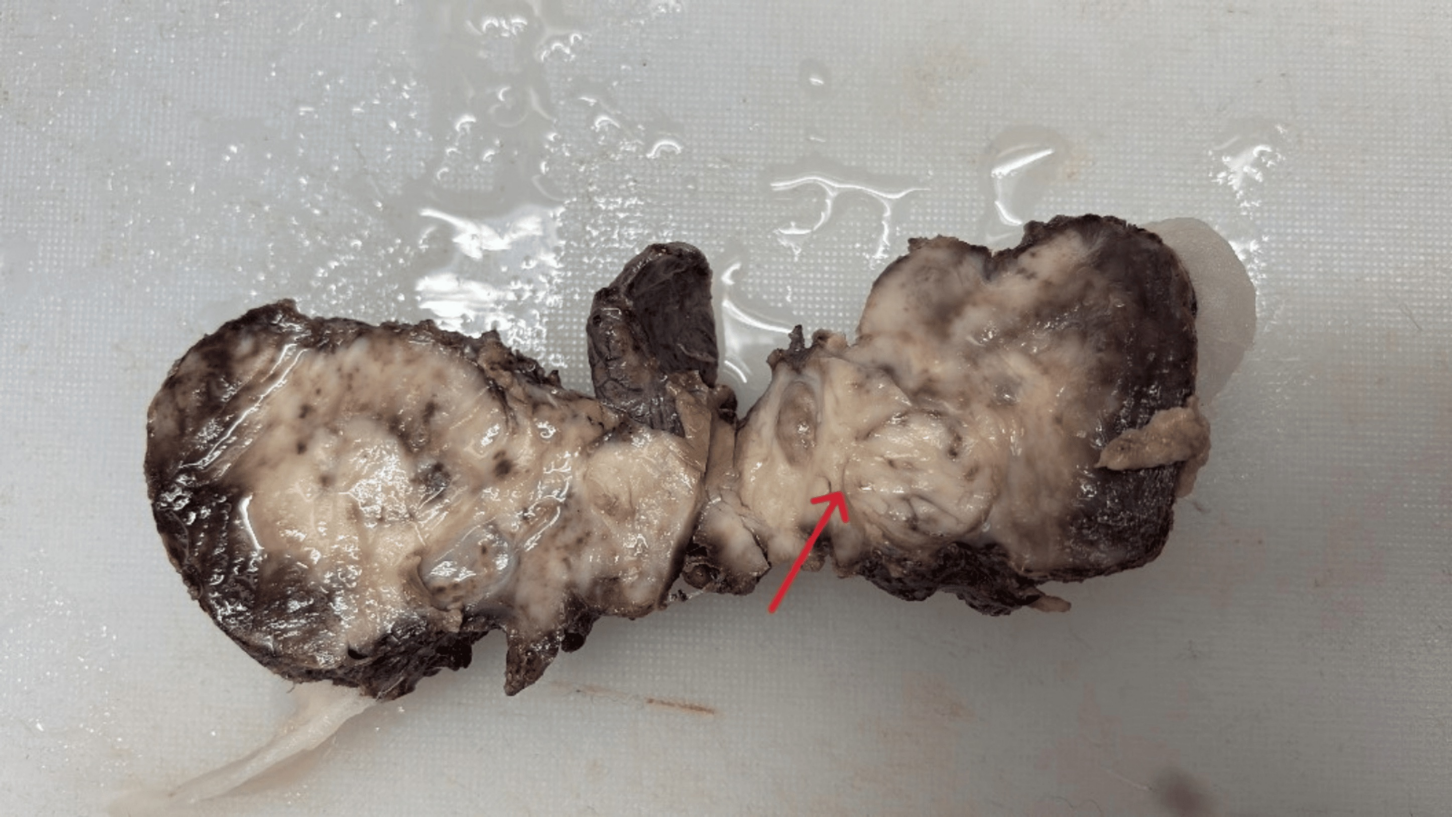
Gross cut section of the thyroid gland showing solid pale yellowish to whitish tissue with infiltrating margin (red arrow)

Based on these findings, a total thyroidectomy with central neck dissection was performed. The specimen was sent for histopathological and immunohistochemical examination. Gross cut section of the thyroid gland showed solid pale yellowish to whitish tissues with infiltrating margin (Figure 2).

**Figure 3:**
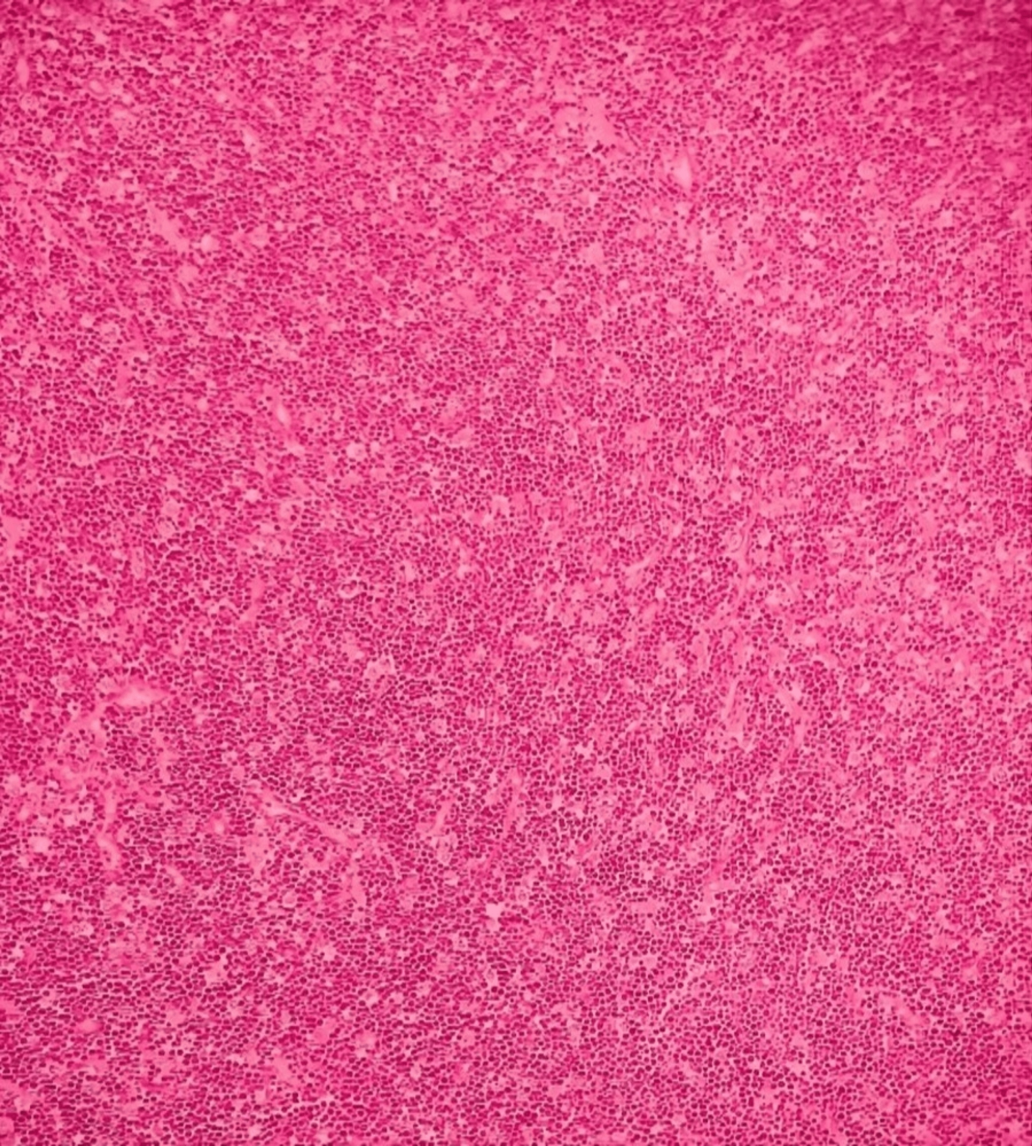
Starry sky appearance (hematoxylin and eosin stain, 10x)

**Figure 4:**
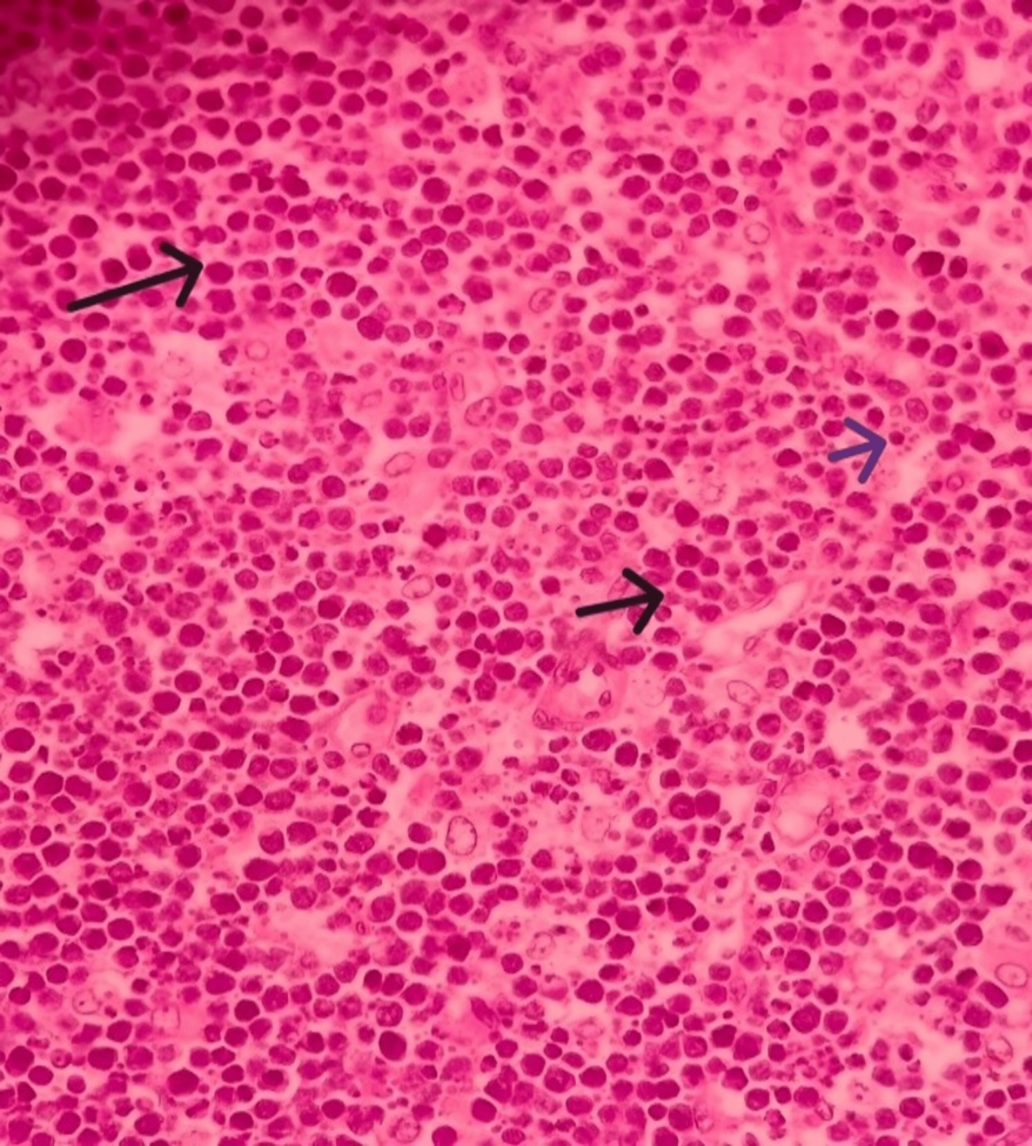
Monotonous intermediate size lymphocytes with finely clumped chromatin (blue arrow) and tingible body macrophages (black arrow) (hematoxylin and eosin stain, 40x)

On histopathological examination, the right lobe of the thyroid showed features of HT. The left lobe and isthmus showed complete effacement of thyroid parenchyma by a monomorphic lymphoid population. The lymphoid cells were small to medium in size with a high nuclear-to-cytoplasmic ratio and stippled chromatin (Figures 3, 4). Gomori methenamine silver stain was suspicious for histoplasma capsulatum. The features suggested two possibilities. One was a lymphoproliferative disorder, and the other was a lymphoplasmacytic reaction in response to suspected histoplasma capsulatum infection. 

**Figure 5:**
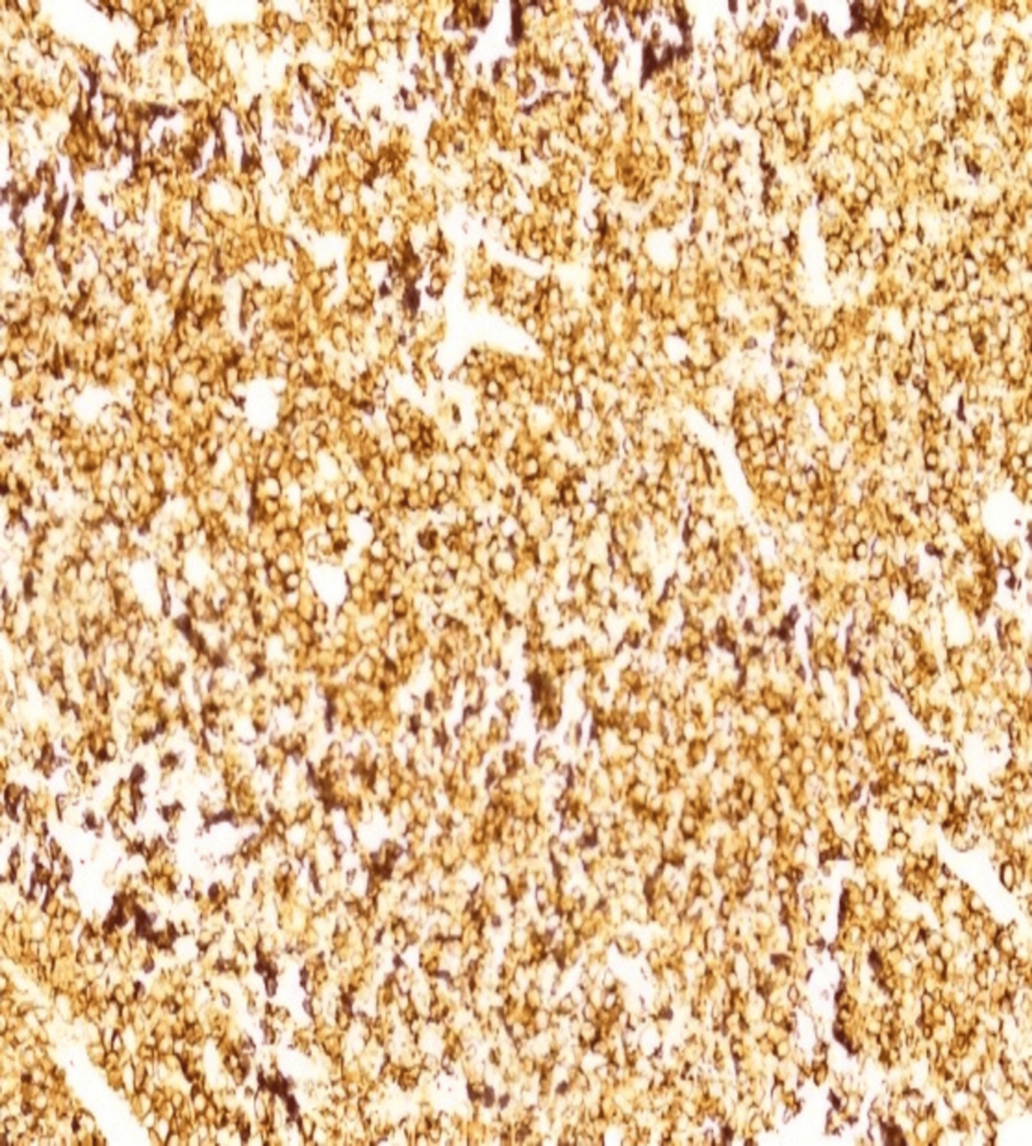
Atypical lymphocytes expressed B-cell markers, including CD20 and co-expressed CD10

Immunohistochemistry showed that atypical lymphocytes expressed B-cell markers, including CD20 and co-expressed CD10. They were negative for BCL-2, cyclin D1, TdT, and CD34 (Figure 5). The Ki-67 proliferation index was high and approached 100%. These findings were consistent with BL. 

A positron emission tomography-computed tomography (PET-CT) scan was performed immediately after the immunohistochemistry confirmation showed postoperative changes after total thyroidectomy in the neck region. No abnormal metabolically active lesion was observed at the operative site. Increased metabolic uptake was noted in both tonsillar fossae, suggesting lymphomatous disease rather than an inflammatory process.

The patient was referred to an oncophysician for further management and was started on the R-CHOEP chemotherapy regimen, which included rituximab, cyclophosphamide, vincristine, adriamycin, etoposide, and prednisolone. Three cycles were administered. The patient was followed up with ultrasound of the neck, abdomen, and pelvis. A repeat PET-CT scan was performed three months after completion of therapy, which showed no metabolically active lesion.

The patient was asymptomatic at follow-up and was advised to continue regular three monthly reviews with the otorhinolaryngology and oncophysician teams.

## Discussion

BL of the thyroid gland is relatively rare. As per a systemic review and meta-analysis performed by Hayashi et al, only 21 cases of primary BL lymphoma of the thyroid have been reported, which mostly occur in adult patients (median age: 39.3 years) with a male predominance (the male-to-female ratio was 13:8) [7].

BL is B-cell non-Hodgkin lymphoma originating from mature germinal center B cells with a short doubling time and high proliferative index and is highly aggressive. Depending on epidemiologic and etiologic characteristics, it has three variants: endemic, sporadic, and immunodeficiency-associated [8]. Primary thyroid disease is extremely uncommon and constitutes less than 3% of primary thyroid lymphomas [9].

The tumor has the characteristic "starry-sky" histology resulting from macrophages holding apoptotic tumor debris, and immunophenotyping is positive for CD20, CD10, and BCL6, with a Ki-67 index of nearly 100%. CD5 and BCL2 are generally negative. Cytogenetically, MYC gene translocation, most often t(8;14)(q24;q32), is diagnostic, while variant rearrangements t(2;8)(p12;q24) and t(8;22)(q24;q11) are rarely encountered [10]. The deregulated MYC expression results in uncontrolled cell-cycle progression and accounts for the tumor's impressive growth kinetics [11].

Primary thyroid BL is typically a rapidly enlarging neck mass in the anterior compartment, often accompanied by airway obstruction or dysphagia. It tends to occur in women with a history of HT, which lends credence to the theory that chronic lymphocytic inflammation leads to lymphoma development [12]. Fine needle aspiration cytology can be deceptive, so tissue biopsy with immunohistochemistry and fluorescence in situ hybridization for MYC rearrangement is required for diagnosis [13].

Management consists of intensive, brief-duration combination chemoimmunotherapy. The CODOX-M/IVAC (cyclophosphamide, vincristine, doxorubicin, high-dose methotrexate alternating with ifosfamide, etoposide, and cytarabine/ifosfamide, etoposide, cytarabine, methotrexate) regimen has produced excellent response rates in adult and pediatric BL. It is the most commonly used protocol [14]. The addition of rituximab to this regimen is beneficial for survival and is now the standard of care [15,16]. Other effective regimens include hyper-CVAD (cyclophosphamide, vincristine, adriamycin, and dexamethasone) and dose-adjusted R-EPOCH, although experience is limited to small series [4]. Active CNS prophylaxis with intrathecal methotrexate and cytarabine is necessary because central nervous system dissemination is highly likely [14].

Quesada et al. studied seven patients with thyroid BL, and most had compressive neck symptoms; with R-Hyper-CVAD or R-EPOCH regimens, most attained complete remission [17]. Recent Indian experience presented by Antony Prestine et al. also showed complete remission following three cycles of R-CODOX-M in a low-risk patient, showing how early diagnosis and protocol-based treatment can be curative [18].

Surgical intervention is restricted to biopsy or airway control; thyroidectomy has no benefit per se, but it is mandatory if initial investigations are not confirmatory of BL. Radiotherapy has a limited role. Prognosis is very good with early initiation of therapy, and survival is over 80% in the limited stage [14].

## Conclusions

Primary BL of the thyroid is a very rare entity. It should always be considered as a differential diagnosis when there is a sudden increase in the size of the thyroid gland, especially in patients with HT. Cytology, histopathology, and immunophenotyping are required for early diagnosis and to differentiate this entity from other thyroid malignancies. Prompt initiation of chemotherapy achieves favorable outcomes.
